# Case report: Surgical treatment of pyloric obstruction with intramural gastric abscess induced by fragmented crystalline foreign materials in a dog

**DOI:** 10.3389/fvets.2024.1427218

**Published:** 2024-08-07

**Authors:** Jihun Kim, Hyunglak Son, Sungin Lee

**Affiliations:** ^1^Department of Veterinary Surgery, College of Veterinary Medicine, Chungbuk National University, Cheongju, Republic of Korea; ^2^Department of Veterinary Surgery, Haemaru Referral Hospital, Seongnam, Republic of Korea

**Keywords:** dogs, Billroth I procedure, foreign material, gastric abscess, pyloric outflow obstruction

## Abstract

An 11-year-old neutered male Maltese presented with a 2-day history of persistent vomiting and lethargy. Abdominal ultrasonography revealed a hypoechoic marginal mass with gastric wall thickening in the pyloric region of the stomach. Computed tomography revealed a non-contrast-enhanced mass in the pyloric antrum causing pyloric outflow obstruction. Imaging studies suggested a tumor and surgical treatment was performed due to the deterioration of the patient’s condition. The pyloric mass was excised, and the stomach and duodenum were anastomosed via pylorectomy and gastroduodenostomy (Billroth I procedure). Postoperatively, the pyloric outflow obstruction resolved, clinical symptoms improved, and no significant complications were observed. Histopathological examination revealed a gastric abscess characterized by a mass-like area with abundant necrosis. Angular fragmented crystalline foreign materials were observed within the lesion. To our knowledge, this is the first reported case of an intramural gastric abscess caused by fragmented crystalline foreign materials in a dog. Although rare, this case highlights the importance of considering gastric abscesses in the differential diagnoses of gastric masses. If the cause of the gastric abscess is an invisible foreign material, postoperative follow-up should be considered to monitor for potential recurrence.

## 1 Introduction

In human medicine, intramural gastric abscess represents a rare form of localized suppurative gastritis characterized by confined pyogenic inflammation of the gastric wall (1). Its rarity is attributed to the bactericidal effect of gastric acid and the abundant blood supply to the stomach (2). Although the exact pathogenesis of intramural gastric abscess remains elusive in human medicine, suspected pathogenic mechanisms include direct microorganism invasion due to gastric mucosal injury (3). Related predispositions include gastric tumors, gastric surgery, endoscopic biopsy, and penetrating mucosal trauma due to foreign bodies (3–6).

Diagnosing intramural gastric abscesses in humans can be challenging, as they mimic stomach cancers in imaging studies, leading to misdiagnosis (4, 7). Although the direct endoscopic demonstration of purulent fluid leakage can confirm the diagnosis, such a discharge may not always be visible, particularly if the abscess resembles a tumor. Furthermore, the rarity of gastric abscess occurrence may also contribute to the diagnostic challenges.

In veterinary medicine, intramural gastric abscesses are exceedingly rare, with only one reported case in a dog at this time (8). This report describes a case where surgical treatment (Billroth I procedure) was performed on a patient presenting with pyloric obstruction suspected of having a tumor based on imaging studies. The surgery was successful, and histopathological examination confirmed that the pyloric lesion was an intramural gastric abscess caused by a fragmented crystalline foreign body.

## 2 Case description

An 11-year-old neutered male Maltese was referred to our hospital with a 2-day history of acute-onset of persistent vomiting and lethargy. On physical examination, the patient had a delayed skin turgor and was found to be 7% dehydration, tachypneic (66 breaths/min), normal blood pressure (110 mmHg), normal heart rate (138 beats/min), and a normal temperature (39.3°C). Serum biochemistry revealed high blood urea nitrogen (30.5 mg/dL; reference range, 8 to 26 mg/dL), elevated total protein (7.7 g/dL; reference range, 5.4 to 7.4 g/dL), hyperglobulinemia (4.6 g/dL; reference range, 2.3 to 4.4 g/dL), elevated alkaline phosphatase (177 U/L; reference range, 15 to 127 U/L), and elevated C-reactive protein (198 mg/L; reference range, 0 to 20 mg/L). Blood gas analysis showed hypokalemia (2.55 mmol/L; reference range, 4.0 to 5.4 mmol/L) and hyponatremia (134 mmol/L; reference range, 142 to 152 mmol/L). A complete blood count indicated leukocytosis (white blood cells, 27.3 K/μL; reference range, 6.0 to 17.0 K/μL).

**Figure 1:**
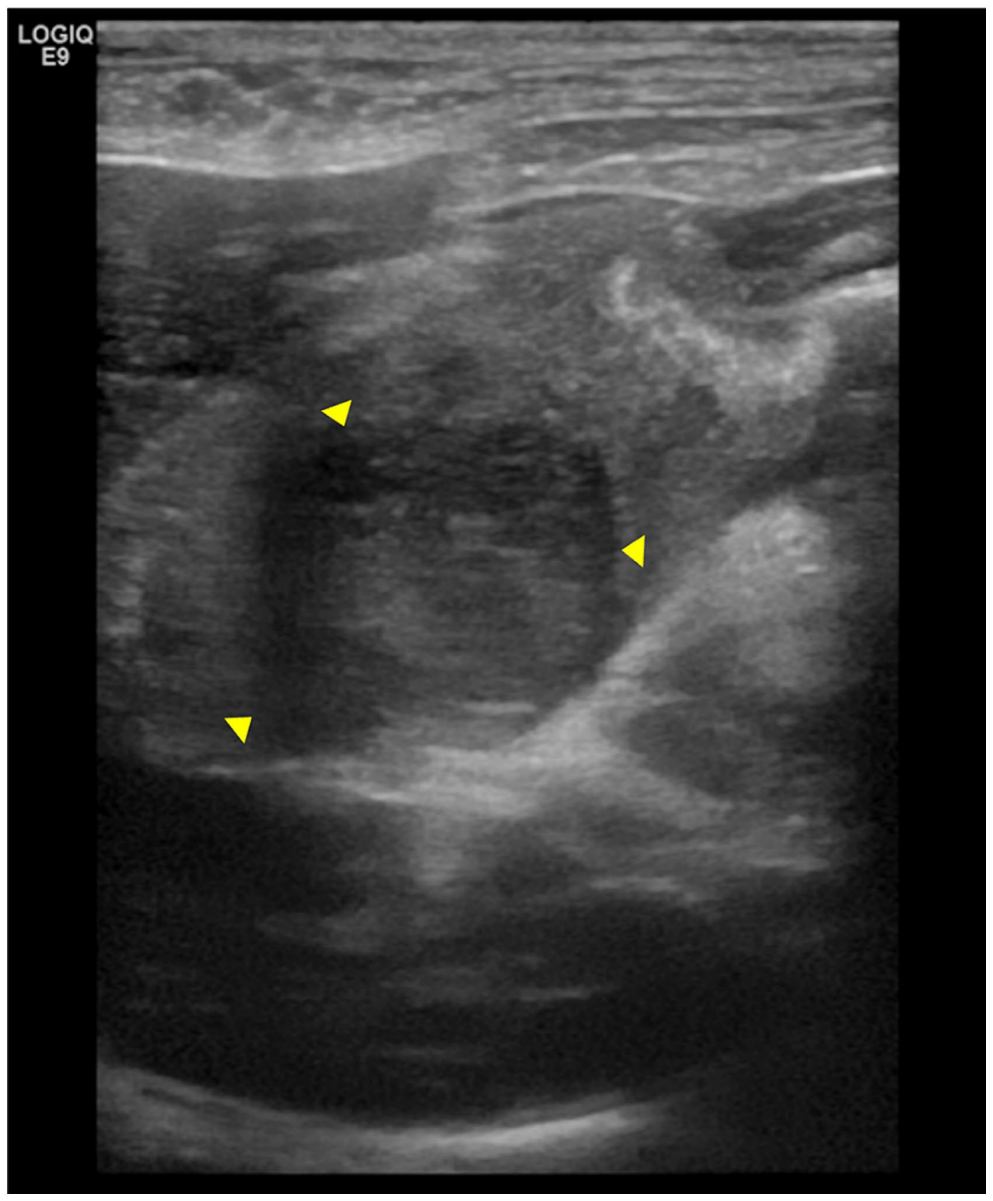
Abdominal ultrasonography images of the pylorus lesion. The arrowheads indicate a mass in the pylorus region, measuring 2.1 cm × 2.2 cm. The mass is surrounded by thickened gastric wall.

Abdominal ultrasonography revealed a 2.1 cm × 2.2 cm hypoechoic, homogeneous, marginal mass in the pyloric region of the stomach (Figure 1). When including the wall thickening lesion around the mass, it measured 2.1 cm × 3.7 cm. Mesenteric echo enhancement was identified around the mass and the distance between the margins of the mass and the common bile duct opening was 0.8–1.2 cm. The mass was causing significant obstruction of the gastric lumen, resulting in gastric dilatation and retention of the intragastric contents. Ultrasound-guided fine-needle aspiration of the gastric mass was performed, and no significant cells were observed on cytology.

**Figure 2:**
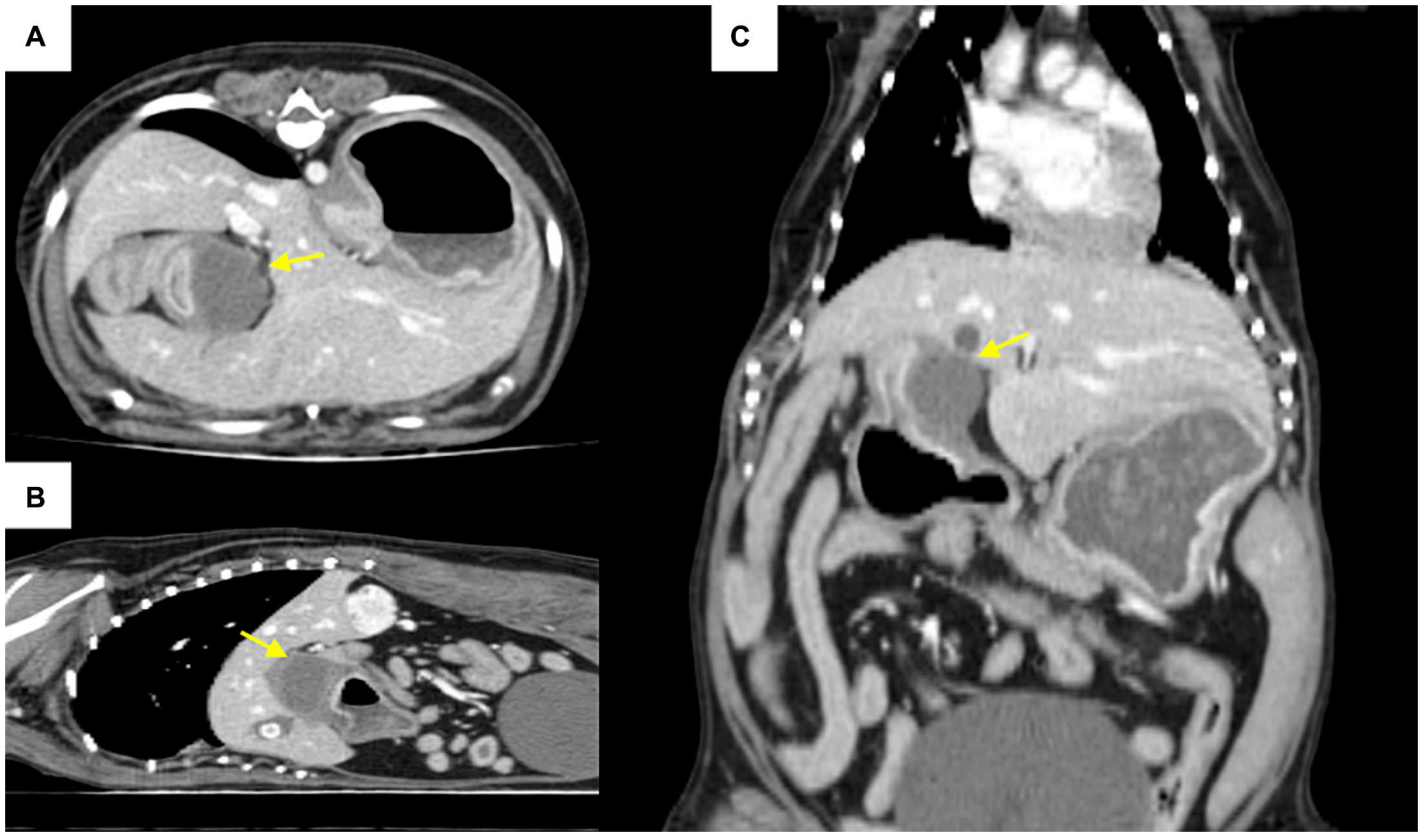
Computed tomography post-contrast scan images **(A)**: axial, **(B)**: coronal, **(C)**: sagittal view. The arrow indicates a mass in the pyloric antrum region that is causing pyloric outflow obstruction.

Computed tomography revealed a focal, well-circumscribed, smoothly marginated mass in the pyloric antrum, which did not show contrast enhancement (Figure 2). On computed tomography and abdominal ultrasonography, the lesion was suspected to be a mass in the pyloric antrum, and the differential diagnoses included leiomyoma, gastrointestinal stromal tumors (GISTs), granuloma, polyp, and malignant tumor. The patient exhibited severe clinical symptoms due to pyloric obstruction. As a result, a surgical approach was considered.

**Figure 3:**
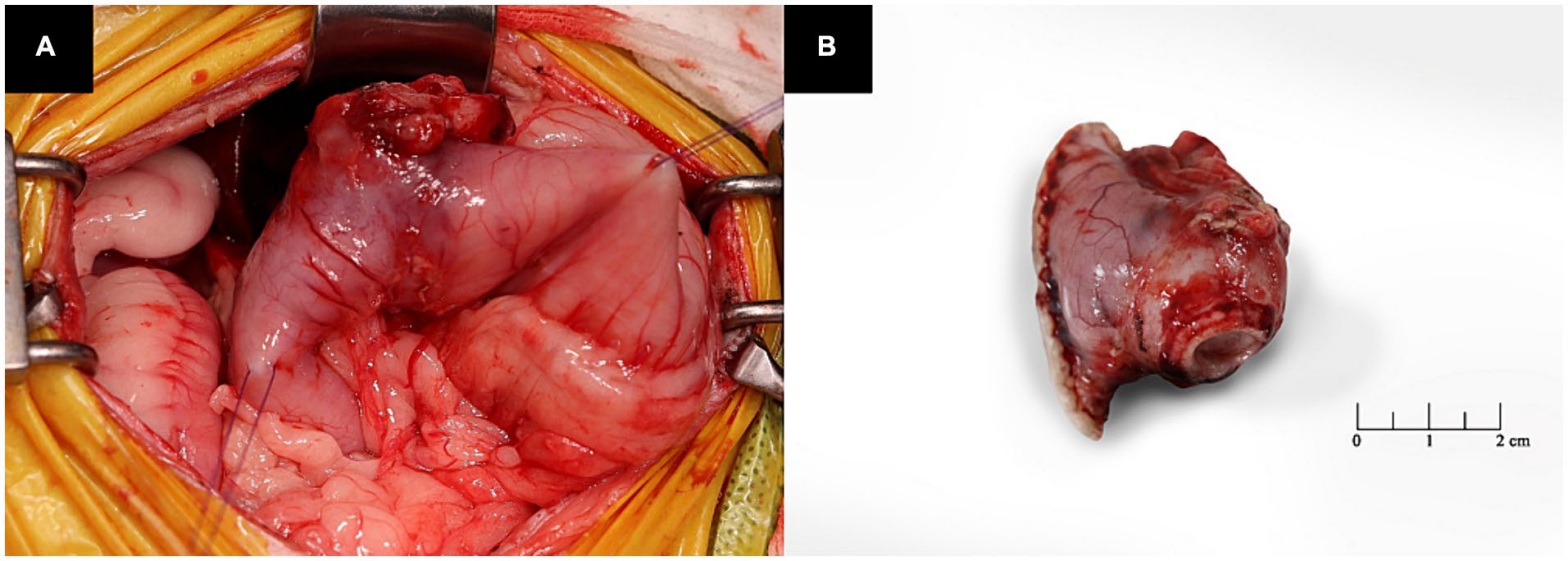
Intraoperative images of the patient. **(A)** The mass is observed in the pyloric antrum. **(B)** The lesion is a resected pyloric mass.

Pylorectomy and gastroduodenostomy were performed. The patient was pre-medicated with intravenous midazolam and fentanyl (0.2 mg/kg and 0.002 mg/kg, respectively), followed by induction of anesthesia with propofol (6 mg/kg, intravenously). Subsequently, endotracheal intubation was performed and anesthesia was maintained using isoflurane in oxygen. A ventral midline abdominal incision was made from the xiphoid to the umbilicus. The greater and lesser omentum and portal triad of the gastroduodenal junction were isolated, and a mass was identified in the pyloric antrum (Figure 3). Stay sutures were placed in the upper section of the duodenum and pyloric antrum using polydioxanone (PDS II) 3 to 0. The gastric wall of the pyloric region was resected using an articulating stapler (Endo GIA; Medtronic Plc, Dublin, Ireland). Duodenal resection was performed using Metzenbaum scissors and Doyen intestinal forceps, followed by stomach anastomosis. To increase the gastric emptying time and minimize dumping syndrome, anastomosis was performed on the dorsal plane of the stomach. The abdominal cavity was lavaged with warm saline before closure. The abdomen was routinely closed. For postoperative management, a jejunal feeding tube (Nasogastric Feeding tube 6Fr × 90 cm; Mila Inc., Kentucky, Ireland) and an active suction drain (Jackson Pratt, Primed, Ireland) were placed.

**Figure 4:**
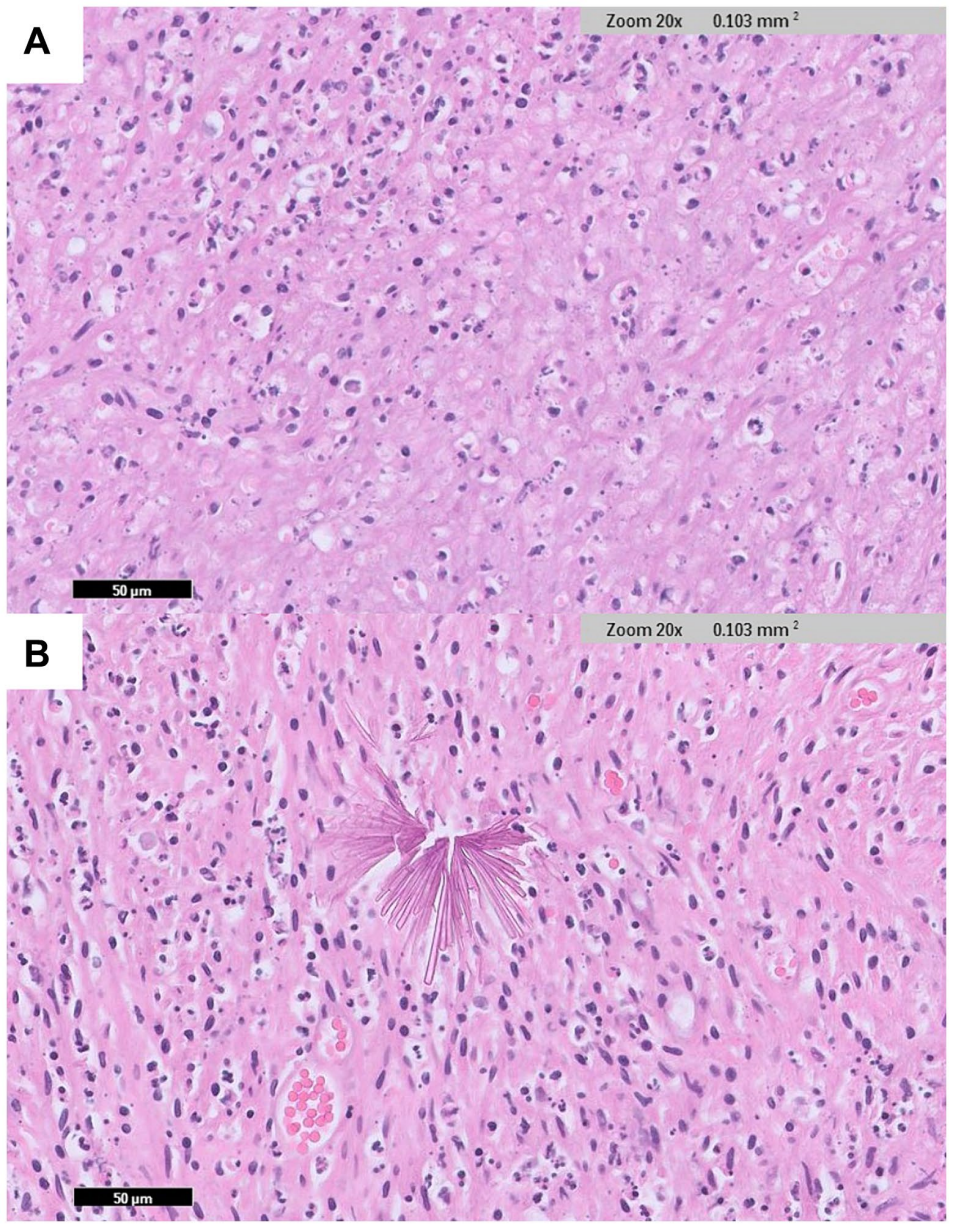
Histopathological examination of the pyloric lesion (IDEXX Laboratories, Westbrook, Maine, United States). **(A)** In the wall of the stomach, an abscess area with abundant necrosis and many degenerate neutrophils are observed (H&E: ×20 magnification). **(B)** Sharply delineated, angular fragment crystalline foreign materials are identified within the lesion (H&E: ×20 magnification).

Histopathological examination of the pyloric mass revealed a gastric mural abscess containing foreign crystalline material. A circumscribed mass-like area with abundant necrosis and numerous degenerate neutrophils was observed in the stomach walls (Figure 4A). Additionally, multifocal, thin, smooth, sharply delineated, refractile appearing eosinophilic to orange, angular fragments indicative of crystalline foreign materials were identified within the lesion (Figure 4B). The mucosal surface was lined with mildly hyperplastic epithelium.

Postoperative medications included maropitant (1 mg/kg, intravenously, q24h), esomeprazole (1 mg/kg, intravenously, q12h), ondansetron (0.5 mg/kg, intravenously, q12h), metoclopramide (0.5 mg/kg, intravenously, q12h), marbofloxacin (2 mg/kg, intravenously, q24h), metronidazole (10 mg/kg, intravenously, q12h), ampicillin-sulbactam (22 mg/kg, intravenously, q8h), and sodium alginate (1 cc/kg, orally, q12h). Pain management was achieved through a constant-rate fentanyl infusion at 0.02ug/kg/min.

Nutritional support was carefully managed post-surgery due to the potential risk of complications. Feeding was initiated 24 h post-surgery via a jejunal tube. Initially, a liquid diet amounting to a quarter of the patient’s resting energy requirements (RER) was administered in six divided doses per day. On the second postoperative day, the amount was increased to half of the RER. On the third postoperative day, feeding was transitioned to a nasogastric feeding tube, delivering the full RER in four divided doses per day. On the fourth day of admission, the patient had recovered voluntary appetite and was fed a wet diet at 1.3 times the RER. Throughout hospitalization, no gastrointestinal symptoms such as vomiting or regurgitation were observed, allowing for a gradual increase in nutritional support. The exudate from the active suction drain gradually decreased and was subsequently removed. With no observed complications, the patient was discharged on postoperative day 5. The patient recovered well after discharge, showing no clinical symptoms. Abdominal ultrasonography follow-up on postoperative day 21 showed resolution of gastric retention and no abnormal findings in the anastomotic region. At 13 months postoperatively, a telephone follow-up with the owner indicated that there was no recurrence of clinical signs.

## 3 Discussion

Suppurative gastritis is classified into three types in human medicine: diffused, localized, and mixed forms (4). The localized form, known as intramural gastric abscess, predominantly involves invasion of the gastric wall, with the pylorus and antrum being the most affected regions. However, gastric abscess formation can also occur throughout the entire stomach (1). In this present case, the intramural gastric abscess was in the pyloric antrum, leading to obstruction of gastric outflow.

In human medicine, differentiating between intramural gastric abscesses and tumors using imaging poses a challenge (4). Similarly, in veterinary medicine, there is no established consensus on how to differentiate gastric tumors from abscesses using imaging studies. An abscess can occur with inflammation of the surrounding tissues; therefore, it can have a similar appearance to cancer and a similar morphology within the tissue. Furthermore, the lack of specific clinical symptoms adds to the diagnostic complexity. Abscesses penetrating adjacent organs or cancerous lesions in an adjacent organ invading the stomach wall to form a tumor-like masses further blur the distinction between malignancy and abscess (4). This confusion can extend to gastric cancer, GISTs, and neuroendocrine neoplasms (7). In one study, malignant mesenchymal cell tumors appeared focal and smoothly marginated on computed tomography and hypoechoic on abdominal ultrasonography (9). In this case, the mass appeared as a focal, well-circumscribed, smoothly marginated tumor on computed tomography and was confirmed as a homogeneous, hypoechoic, marginal mass on abdominal ultrasonography. Fluid echo and foreign body echo were not observed, and considering the appearance of the tumor, we suspected a gastric tumor rather than an abscess.

In the present case, no foreign bodies were observed on computed tomography, abdominal ultrasonography, or surgery. However, crystalline foreign materials were identified on histopathological examination along with the abscess, with no neoplastic cells observed. Therefore, the patient was diagnosed with a foreign body-induced gastric abscess. In human medical literature, visible penetrating foreign bodies often cause intramural gastric abscess (10–12). Although one study reported an abscess caused by small, non-visible fragment materials, the fragmented crystalline materials observed in the histopathology of our case have not been reported in a similar form in either human or veterinary medicine, to the best of our knowledge (13). These crystalline foreign materials were suspected to originate from finely fragmented debris, such as ingested foreign objects or environmental substances. Even in the absence of a history of foreign body ingestion or trauma, and with no foreign body identified on imaging, the possibility of a foreign body causing a gastric abscess should be considered when a gastric abscess is present.

In the human medical literature, treatment modalities for intramural gastric abscess include surgery, endoscopic drainage, percutaneous drainage, and drainage without antibiotics alone (3, 14). Gastrectomy with antibiotics has been considered the recommended treatment, and recently endoscopic drainage represents a less invasive and effective treatment. If a foreign body is identified in an abscess, either through imaging or visually, it can be removed, followed by drainage. However, there have been cases where foreign bodies were not detected in the abscess but histopathological examination confirmed their presence (13). Additionally, there have been cases where foreign bodies were present but undetected and the abscess continued to recur when only drainage was performed, resulting in multiple surgeries (14). Therefore, if an abscess is caused by a foreign body that cannot be visually identified, surgical intervention becomes a viable option. This is because drainage alone can result in abscess recurrence if the foreign body fragment is not removed.

In this case, treatment planning initially leaned towards addressing the suspected tumor, given the absence of indications suggestive of an abscess on imaging studies. Furthermore, the lesion was large and located in the pyloric antrum, obstructing gastric outflow, and the clinical symptoms persisted. Therefore, considering the size, location, and clinical signs of the mass, prompt surgical intervention was recommended. Because the obstruction was severe and suspected to be tumor-induced, pyloromyotomy and pyloroplasty were unlikely to improve the outflow obstruction. Therefore, pylorectomy and gastroduodenostomy (Billroth I procedure) were performed. Surgical treatment successfully managed the pyloric obstruction attributed to a gastric abscess caused by a crystalline foreign material. Post-surgical follow-up revealed an improvement in clinical symptoms with no complications identified.

In conclusion, this is the first report of an intramural gastric abscess caused by crystalline foreign materials in a dog. Although rare, the possibility of gastric abscess should not be ruled out if a gastric mass is identified. The possibility that the abscess is not detectable on imaging examination should also be considered. If unseen microscopic foreign material remains, the abscess may continue to recur despite drainage, and follow-up should be performed to fully assess recurrence. Furthermore, histopathological examination post-surgery is essential for accurate diagnosis and planning of future treatment strategies.

## Data availability statement

The original contributions presented in the study are included in the article/Supplementary material, further inquiries can be directed to the corresponding author.

## Ethics statement

Ethical approval was not required for the studies involving animals in accordance with the local legislation and institutional requirements because this case report is research for therapeutic purposes and the owner's consent has been obtained. Written informed consent was obtained from the owners for the participation of their animals in this study.

## Author contributions

JK: Conceptualization, Data curation, Formal analysis, Software, Visualization, Writing – original draft, Writing – review & editing. HS: Conceptualization, Data curation, Resources, Software, Writing – review & editing. SL: Conceptualization, Funding acquisition, Supervision, Validation, Writing – review & editing.
